# Disruption of Her2-Induced PD-L1 Inhibits Tumor Cell Immune Evasion in Patient-Derived Gastric Cancer Organoids

**DOI:** 10.3390/cancers13246158

**Published:** 2021-12-07

**Authors:** Jayati Chakrabarti, Vivien Koh, Nina Steele, Jennifer Hawkins, Yoshiaki Ito, Juanita L. Merchant, Jiang Wang, Michael A. Helmrath, Syed A Ahmad, Jimmy Bok Yan So, Wei Peng Yong, Yana Zavros

**Affiliations:** 1 Department of Cellular and Molecular Medicine, College of Medicine, University of Arizona, 1501 N Campbell Avenue, Tucson, AZ 85719, USA; chakraj@email.arizona.edu; 2 National University Cancer Institute Singapore, National University Health System, Singapore 119228, Singapore; csiv92@visitor.nus.edu.sg (V.K.); jimmy_so@nuhs.edu.sg (J.B.Y.S.); wei_peng_yong@nuhs.edu.sg (W.P.Y.); 3 Cancer Science Institute of Singapore, National University of Singapore, Singapore 117599, Singapore; yoshi_ito@nus.edu.sg; 4 Department of Cell and Developmental Biology, University of Michigan, Ann Arbor, MI 48109, USA; steelen@med.umich.edu; 5 Department of Surgery, University of Michigan, Ann Arbor, MI 48109, USA; 6 Department of Pediatric Surgery, Cincinnati Children’s Hospital Medical Center, Cincinnati, OH 45229, USA; Jennifer.Hawkins@cchmc.org (J.H.); Michael.Helmrath@cchmc.org (M.A.H.); 7 Department of Gastroenterology and Hepatology, University of Arizona College of Medicine, Tucson, AZ 85719, USA; jmerchant@deptofmed.arizona.edu; 8 Department of Pathology and Laboratory Medicine, University of Cincinnati College of Medicine, Cincinnati, OH 45267, USA; Jiang.Wang@uc.edu; 9 Department of Surgery, University of Cincinnati Cancer Institute, Cincinnati, OH 45267, USA; ahmadsy@ucmail.uc.edu; 10 Department of Surgery, Yong Loo Lin School of Medicine, National University of Singapore, Singapore 117597, Singapore

**Keywords:** organoid-immune cell co-culture, PD-L1, myeloid-derived suppressor cells

## Abstract

**Simple Summary:**

HER2 may contribute to immune evasion in gastric cancer that is associated with PD-L1 expression. Autologous organoid/immune cell co-cultures serve as an appropriate in vitro model to study the effects of anti-HER2 targeted therapy in combination with anti-PD1 immune checkpoint inhibition and may be used as an ex vivo tool for precision medicine.

**Abstract:**

(1) Background: The expression of programmed death-ligand 1 (PD-L1), which interacts with programmed cell death protein 1 (PD-1) on cytotoxic T lymphocytes (CTLs), enables tumors to escape immunosurveillance. The PD-1/PD-L1 interaction results in the inhibition of CTL proliferation, and effector function, thus promoting tumor cell evasion from immunosurveillance and cancer persistence. Despite 40% of gastric cancer patients exhibiting PD-L1 expression, only a small subset of patients responds to immunotherapy. Human epidermal growth factor receptor2 (HER2) is one of the critical regulators of several solid tumors, including metastatic gastric cancer. Although half of PD-L1-positive gastric tumors co-express HER2, crosstalk between HER2 and PD-1/PD-L1 in gastric cancer remains undetermined. (2) Methods: Human gastric cancer organoids (huTGOs) were generated from biopsied or resected tissues and co-cultured with CTLs and myeloid-derived suppressor cells (MDSCs). Digital Spatial Profiling (DSP) was performed on FFPE tissue microarrays of numerous gastric cancer patients to examine the protein expression of immune markers. (3) Results: Knockdown of HER2 in PD-L1/HER2-positive huTGOs led to a concomitant decrease in PD-L1 expression. Similarly, in huTGOs/immune cell co-cultures, PD-L1 expression decreased in huTGOs and was correlated with an increase in CTL proliferation which enhanced huTGO death. Treatment with Nivolumab exhibited similar effects. However, a combinatorial treatment with Mubritinib and Nivolumab was unable to inhibit HER2 expression in co-cultures containing MDSCs. (4) Conclusions: Our study suggested that co-expression of HER2 and PD-L1 may contribute to tumor cell immune evasion. In addition, autologous organoid/immune cell co-cultures can be exploited to effectively screen responses to a combination of anti-HER2 and immunotherapy to tailor treatment for gastric cancer patients.

## 1. Introduction

Gastric cancer is the third most prevalent for cancer-related mortality [1,2]. While there is a relatively low incidence of gastric cancer in the United States due to early diagnosis and treatment of *Helicobacter pylori* (*H. pylori*) [3], the 5-year survival rate remains at a dismal 10–30% [4]. Tumors evade immunosurveillance by expressing programmed death-ligand 1 (PD-L1) that interacts with programed cell death protein 1 (PD-1) on CD8+ cytotoxic T lymphocytes and subsequently inhibits immune cell proliferation and effector function [5,6,7]. Inhibition of the PD-1/PD-L1 axis has led to effective therapeutic responses in different solid cancers, such as melanoma, renal and non-small cell lung cancer [8,9,10,11]. Several anti-PD-1 antibodies are currently being tested in ongoing clinical trials for the treatment of gastric cancer. Despite the fact that 35–40% of gastric tumors express PD-L1, immunotherapy responses are relatively low [12,13,14,15,16]. In addition, PD-L1 expression does not consistently predict the survival benefit of anti-PD-1 therapy [17]. This evidence drives the need to develop a preclinical in vitro model system to characterize the tumor-immune microenvironment and test for immunotherapy responses of individual patients.

The amplification and overexpression of HER2 were first discovered in breast cancer and are well-known to be significantly correlated with a worse prognosis [18]. HER2 targeted therapy has recently been introduced for the treatment of advanced gastric cancer [19]. As such, determination of the HER2 status is critical to select patients who will benefit from this treatment. Trastuzumab, a monoclonal anti-HER2 antibody, has demonstrated success in treating patients with HER2-positive advanced gastric cancer and led to a rapidly increasing clinical demand for HER2 assessment [20]. However, HER2 testing in gastric cancer differs from that in breast cancer due to inherent differences in the tumor biology, an intratumoral heterogeneity of HER2 expression, as well as an incomplete staining on the membranes that is frequently detected in gastric tumors [21]. There is no general agreement on the mechanisms of action of trastuzumab. Preliminary studies showed that trastuzumab prevents HER2 dimerization with other isoforms and stimulates endocytosis to induce cell-mediated immunity [22]. HER2 possibly regulates stomach PD-L1 aberrant expression [23] and the combination therapy of anti-HER2 and anti-PD-1 has demonstrated synergistic antitumor activity in animal models [24,25].

Randomized clinical findings have distinctly indicated that multimodality therapy is superior to surgery alone for gastric cancer treatment to improve overall survival and minimize recurrence [26]. However, the current problem with the treatment of gastric cancer is the absence of a dependable model to tailor the most effective treatment for each patient.

## 2. Materials and Methods

### 2.1. Selection of the Patient Population for the Study

Patients who underwent a total or partial gastrectomy with a confirmed clinicopathological diagnosis for gastric cancer were enrolled in this study at the National University Hospital of Singapore. Informed consent was obtained from all eligible subjects who were older than 21 years and naïve for radiotherapy and chemotherapy prior to surgical procedures. Patients having progressive metastasis and other underlying medical ailments were excluded from the study. Study procedures were thoroughly evaluated and permitted by the National Healthcare Group Domain Specific Review Board (NHG DSRB; Reference Numbers: 2005/00440, 2016/00059) following the institutional laws and regulations in accordance with the Declaration of Helsinki and International Conference on Harmonization and Good Clinical Practice guidelines. Likewise, tumor tissues were collected from patients having surgery for gastric cancer at the University of Cincinnati (IRB protocol number: 2015-5537, University of Cincinnati; or IRB protocol number: 1912208231R001) or Endoscopic ultrasound (EUS) guided biopsies (University of Arizona Human Subjects Protection Program; IRB protocol number: 1099985869R001, University of Arizona Human Subjects Protection Program TARGHETS). Patient demographics along with the clinicopathological diagnosis reported in this study are summarized in Table 1.

### 2.2. Generation of Human Resected Tumor Derived Organoids

Organoids were derived from the resected gastric tumor tissues following a formerly published protocol [27,28,29,30]. To summarize, tumor tissue was collected in ice-cold antibiotic enriched DPBS (Dulbecco’s phosphate buffered saline, exclusive of calcium or magnesium, supplemented with 1% penicillin/streptomycin, 1% kanamycin and 0.25 mg/mL Amphotericin B/10 mg/mL Gentamicin). The tissue was cut into smaller pieces, incubated for 15 min in 10 mL EDTA stripping buffer (HBSS with 20% FCS, 1% penicillin/streptomycin, HEPES and EDTA) at 37 °C in a rotating shaker, washed twice with antibiotic supplemented Dulbecco’s Modified Eagle’s medium (DMEM) and incubated with 10 mL collagenase A-hyaluronic acid buffer at 37 °C in a rotating shaker for 15–30 min. Processed tissue was then strained through a 40 μm filter, centrifuged at 400× *g* for 5 min, and washed with cold DPBS. The pellet was resuspended in the required volume of Matrigel™ (Corning, NY, USA) and seeded into culture plates. The Matrigel dome was overlaid with gastric organoid growth medium (Table 2) and maintained at 37 °C in a CO_2_ incubator. The medium was replaced every 3–4 days.

### 2.3. Generation of PBMCs (Peripheral Blood Mononuclear Cells) from Human Blood

Autologous whole blood from the same gastric cancer patients was processed for the isolation of PBMCs using a Lymphoprep™ (STEMCELL Technologies, Vancouver, Canada) density gradient medium. Following the manufacturer’s protocol, the blood was diluted (50% *v*/*v*) in blood wash buffer (1% FCS in DPBS) and added to Lymphoprep™ in a SepMate^TM^ 50-IVD (STEMCELL Technologies) 50 mL tube. The blood was centrifuged at 1200× *g* for 10 min and the leukocyte-enriched supernatant was diluted 50% *v*/*v* with the blood wash and centrifuged at 300 g for 8 min. The supernatant was removed, and the pellet was resuspended in 10 mL of the blood wash, followed by a centrifugation at 120× *g* for 10 min in order to separate the leukocytes from the platelets. The pellet was either resuspended in freezing medium (45% RPMI, 45% human serum and 10% DMSO) and stored at −80 °C or, used for growing specific immune cells.

### 2.4. Isolation and Culture of Human Immune Cells

Human dendritic cells, cytotoxic T lymphocytes (CTLs) and myeloid-derived suppressor cells (MDSCs) were isolated from PBMCs and cultured as previously published [30].

#### 2.4.1. Dendritic Cells (DCs)

PBMCs were cultured in AIM V medium containing 10% human serum AB (Gemini Bio, West Sacramento, CA), 50 μM β-mercaptoethanol, 800 U/mL granulocyte-macrophage colony-stimulating factor (GM-CSF) and 500 U/mL interleukin 4 (IL-4) for 3 days following a previously published protocol [31]. The cultures were then matured with fresh medium additionally supplemented with 5 ng/mL tumor necrosis factor α (TNF-α), 5 ng/mL interleukin 1β (IL-1β), 150 ng/mL interleukin 6 (IL-6) and 1 μg/mL prostaglandin E2 (PGE2), and further maintained for 24 h.

#### 2.4.2. Cytotoxic T Lymphocytes (CTLs)

CD8+ T cells were generated from PBMCs using the EasySep Enrichment Kit (STEMCELL Technologies) following the manufacturer’s protocol. Briefly, PBMCs were incubated with 50 μL/mL of the Enrichment Cocktail for 10 min at room temperature, followed by the addition of 150 μL/mL of Magnetic Particles and incubation for 5 min. The cocktail and the magnetic particle complex were then diluted with EasySep Buffer (STEMCELL Technologies) and positioned into an EasySep Magnet (STEMCELL Technologies). The separated CD8+ T cell suspension was collected into a new tube, centrifuged at 300× *g* for 5 min and cultured as in the previously published protocol [30].

#### 2.4.3. Myeloid-Derived Suppressor Cells (MDSCs)

PBMCs were grown for 7 days in a monocyte culture medium, supplemented with appropriate cytokines for MDSC differentiation (AIM V contained 10 ng/mL IL-1β, 10 ng/mL IL-6, 1 μg/mL PGE2, 2 ng/mL transforming growth factor beta 1 (TGF-β1), 10 ng/mL TNF-α, 10 ng/mL vascular endothelial growth factor (VEGF) and 10 ng/mL GM-CSF) plus 50% conditioned medium obtained from autologous organoid cultures (huTGO^CM^) [32].

The immune cells were collected and used for respective downstream co-culture experiments. All reagents were purchased from Thermo Fisher Scientific, unless otherwise stated.

### 2.5. Establishment of Organoids and Immune Cells in Co-Culture

Autologous organoid/immune cell co-cultures were established as described previously [30,33]. Dendritic cells were initially pulsed with huTGO^CM^, followed by co-culturing them with CTLs for 24 h. CTLs were then harvested and labelled with 5 µM carboxyfluorescein diacetate succinimidyl ester (CFSE; BioLegend, San Diego, CA, USA) at 37 °C for 20 min to monitor cellular proliferation. huTGOs were then harvested, combined with CFSE-labelled CTLs, resuspended in the required quantity of Matrigel and seeded into multiwell plates. MDSCs were mixed with huTGOs and CFSE-labelled CTLs and seeded according to the experimental need. Co-cultures were sustained in gastric huTGOs culture medium at 37 °C in 5% CO_2_.

The following groups of drug treatment conditions were designed: (1) vehicle-treated huTGOs and CTL co-cultures, (2) huTGOs and CTL co-cultures treated with 0.5 µg/mL Nivolumab (anti-PD-1; Selleckchem, Houston, TX) or (3) 10 µM Mubritinib (Selleckchem, S2216) alone, (4) co-cultures of huTGOs, CTLs and MDSCs treated with Nivolumab or (5) Mubritinib alone or combined with 10 µM Cabozantinib. Organoid-immune cell co-cultures were treated for 48 h and observed using brightfield microscopy (Nikon Spinning disk Ti_2_ Eclipse inverted confocal microscope, Nikon Corporation, Tokyo, Japan).

Regions of interest (ROIs) were marked manually surrounding each organoid for each treatment group and an “automated measurement” of density, area and perimeter was analyzed using the Nikon Elements (NIS-AR) Software Version 5.21.02. The density/area was computed based on the squared perimeter (mm^2^). The circularity was calculated with the formula, (4 × *pi* × area)/perimeter^2^. Variations in the density were analyzed per area/squared perimeter (mm^2^). The Nuclear Area Factor (NAF) was calculated using the product of the area and circularity measurement using Microsoft excel software.

### 2.6. Orthotopic Transplantation of Gastric Organoids

The orthotopic transplantation surgery of huTGOs was performed in NOD scid gamma (NSG) mice. An initial induction of acetic acid injury was performed followed by transplantation of approximately 500 organoids within the submucosa of the injured site following our established method [27]. All animal studies conducted were approved by the animal ethics committee of the University of Arizona (Protocol Number: 19-571).

### 2.7. Knocking down of ERBB2 (HER2) Expression in Gastric Tumor-Derived Organoids

Mature huTGOs were collected in ice-cold DPBS, centrifuged at 400× *g* for 5 min, and the pellet was resuspended in Accutase. The organoids were incubated at 37 °C for 10–12 min, and gently syringed (26G needle) 10 times. Prewarmed complete growth medium was added to neutralize the Accutase before centrifuging at 400× *g* for 5 min; then the pellet was resuspended in 1 mL 3D growth medium. The empty vector (EV) or the shRNA lentiviral particles (Sigma-Aldrich) were added to the cells using the appropriate MOI unit along with 8 mg/mL hexadimethrine bromide, and incubated overnight at 37 °C. An equal volume of fresh medium was added to the cells and they were centrifuged at 400× *g* for 5 min. Cells were resuspended in Matrigel and plated in 12-well tissue culture plates. A 1mL volume of 3D gastric growth medium was added to each well after 13 min of incubation at 37 °C. Three days later the organoids were selected by adding puromycin (Thermo Fisher Scientific) into the culture. The control (EV) and knockdown (KD) cells were grown for at least 7–10 days. The efficiency of the knocking down of the organoids was tested either using an immunofluorescence and/or Western blot technique.

### 2.8. Immunofluorescent Staining of Whole-Mount Organoids

The immunofluorescence staining was processed using a previously published method [28]. The organoids were fixed by adding 3.7% paraformaldehyde, 200 μL/well for 20 min at room temperature. huTGOs were then washed with 200 μL/well DPBS (Fisher Scientific) for 5 min at room temperature (RT). The organoids were permeabilized for 20 min at RT with 200 μL/well 0.5% Triton X-100 in PBS (PBST). The organoids were washed with 200 μL/well 0.01% PBST for 5 min at RT, followed by blocking for 1 h at RT with 2% normal donkey serum (Jackson Immuno Research, West Grove, PA, USA) diluted in 0.01% PBST, and the incubation with specific primary antibodies, diluted in 0.01% PBST, was at 4 °C for 16–18 h. The organoids were washed for 5 min at RT with 0.01% PBST and incubated under dark conditions for 1 h at RT with the secondary antibody and Hoechst (diluted 1:1000 in 0.01% PBST), diluted in 0.01% PBST. The organoids were then washed for 5 min at RT with 0.01% PBST and stored in DPBS. The primary antibodies used were rabbit anti-PD-L1 (Novus Biologicals, Littleton, CO, USA, NBP1-76769) reactive to human and mouse, and human-specific mouse anti-HER2 (Novus Biologicals, NBP2-01152) with a 1:100 dilution for both.

### 2.9. Immunohistochemistry

Gastric tumor tissues were fixed in 4% paraformaldehyde, embedded in paraffin and sectioned (5 microns). After deparaffinization and antigen retrieval (Antigen Unmasking Solution, Vector Laboratories, Burlingame, CA, USA), endogenous peroxidase activity was blocked using 0.3% hydrogen peroxide/methanol for 20 min. Slides were then blocked with 20% goat or horse serum (ImmPRESS^TM^ HRP Anti-Goat IgG or Anti-rabbit IgG reagent kit, Vector Laboratories) for 20 min at RT, then incubated with either the goat anti-PD-L1 (Novus Biologicals, NB300-903) or rabbit anti-HER2 (Abcam, Cambridge, United Kingdom, 134182) antibody (1:100 dilution) overnight at 4 °C. Tumor tissue sections were then incubated with anti-goat or anti-mouse ImmPRESS Ig (Vector Laboratories) for 30 min at RT. The slides were then incubated with a peroxidase substrate solution from the DAB Peroxidase (HRP) Substrate Kit (Vector Laboratories) and the color intensity of the PD-L1 or HER2 staining was monitored under a brightfield microscope. The slides were mounted with Permount (Fisher Scientific, Pittsburgh, PA, USA) and visualized by light microscopy.

### 2.10. Flow Cytometryic Analysis

After 48 h of treatment, the cell co-cultures were incubated in Accutase (at 37 °C for 15 min) to dissociate huTGOs into single cells as previously published [30]. All cells were collected by centrifuging at 300× *g* for 5 min, then resuspended in 100 µL of diluted Zombie UV dye (1:100 in PBS) and stained for 15 min at RT. The cells were incubated at 4 °C for 30 min with fluorochrome-conjugated antibodies specific for CD8a, CD14, CD15, CD11b, CD33, CD137, EpCAM, granzyme B, Perforin, HLA-DR, and PD-L1 (1:100 dilution, all from BioLegend), diluted in 100 μL cell staining buffer. Cells were washed with cell staining buffer (BioLegend) and incubated with the Cytofix/Cytoperm Fixation/Permeabilization Buffer (BD Biosciences, Franklin Lakes, NJ, USA) for 20 min at 4 °C. Cells were then washed and resuspended in 100 µL of cell staining buffer and stained at 4 °C for 30 min with fluorochrome-conjugated intracellular antibodies specific for perforin, IL-2 and interferon-gamma (IFN-g) (both from BioLegend) diluted in 100 µL cell staining buffer. Cells were washed, resuspended in 300 µL of cell staining buffer, filtered, and then analyzed on an LSRII system (BD Biosciences). An unstained cell sample and single stained beads for each antibody were used as gating controls. Data were analyzed using FlowJo software (BD Biosciences).

### 2.11. Western Blot Analysis of huTGOs

Organoids were harvested using ice-cold DPBS at each experimental timepoint, pelleted at 400× *g* for 5 min, and lysed using phosphatase-free lysis buffer. Protein concentrations were determined using the Bradford assay (Thermo Fisher Scientific). Lysates were first diluted with 4× Laemmli sample buffer containing β-mercaptoethanol (Bio-Rad Laboratories, Hercules, CA) and then loaded onto 4–20% Tris-Glycine gradient gels (Thermo Fisher Scientific). Gels were electrophoresed at 80 V for 3 h and proteins were transferred to 0.45 μM nitrocellulose membranes (Whatman Protran, Maidstone, United Kingdom) at 105 V for 1.5 h at 4 °C. Membranes were blocked with KPL Detector Block Solution (Kirkegaard & Perry Laboratories, Gaithersburg, MD) for 1 h at room temperature, probed with specific primary antibodies overnight at 4 °C, then incubated with the appropriate secondary antibodies for 1 h at room temperature. The primary antibodies used were anti-AKT, anti-phospho AKT, anti-ERK, anti-phospho ERK, anti-HER2, anti-S6K and anti-phospho S6 (all from Cell Signaling Technology, Danvers, MA, USA) each at a 1:1000 dilution and anti-GAPDH (Merck Millipore, Burlington, MA) at a 1:2000 dilution. The secondary antibodies used were anti-mouse and anti-rabbit Alexa Fluor 680 (Thermo Fisher Scientific) each at a 1:1000 dilution. The blots were imaged using a scanning densitometer (Odyssey Infrared Imaging System; LI-COR Biosciences, Lincoln, NE, USA) and analyzed with Odyssey Infrared Imaging Software. The densitometric analysis was executed using ImageJ software—NIH and the ratio against GAPDH or the respective total protein was plotted as violin plots.

### 2.12. Establishment of an HER2 Knockdown (KD) Gastric Tumor Spheroid and Immune Cell Co-Cultures Using AggreWell™ Microwell Plates

AggreWell™ 800 microwell plates (STEMCELL Technologies, 24 well) were used to generate cell aggregates/spheroids. Each plate contains 300 microwells (800 μm per well), enabling the production of large numbers of spheroids. The spheroids (EV and HER2 KD) were generated following the manufacturer’s protocol: 500 mL anti-adherence rinsing solution (STEMCELL Technologies) was added to each well. The plate was centrifuged at 1300× *g* for 5 min and washed with prewarmed basal medium. A 1 mL volume of complete medium was added to each well immediately. The EV and HER2 KD huTGOs were collected in ice-cold DPBS, centrifuged at 400× *g* for 5 min, the supernatant was discarded carefully, and the pellet was incubated with Accutase at 37 °C for 10–12 min. huTGOs were gently syringed (26G needle) 10 times and prewarmed complete growth medium was added to the tube. Organoids were centrifuged at 400× *g* for 5 min and resuspended in complete medium. Cells were pipetted gently up and down several times and equally distributed to each well (4000 cells/mL/well). The AggreWell™ plate was then immediately centrifuged at 100× *g* for 3 min. The cells were cultured for 72 h until the organoids reformed a spherical shape. CTLs with or without MDSCs were then added into the appropriate wells and the culture plate was centrifuged at 100× *g* for 3 min. Following this, the co-cultures were treated with Nivolumab, Mubritinib and/or Cabozantinib according to the experimental conditions and imaged at 24, 48 and 72 h after treatment by brightfield microscopy (Nikon Spinning disk confocal microscope). Images were analyzed using Nikon Element software based on changes in the area of each experimental condition at each timepoint by assigning ROIs to individual spheroids. In a separate series of experiments, spheroids were treated with the mTOR inhibitor Everolimus (SIGMA, SML2282, 2 ng/mL). The area, perimeter and density were measured at 0 and 72 h after treatment. The summarized data was computed as area ± SEM and presented as a histogram using GraphPad Prism software.

### 2.13. Morphology-Driven High-Plex Digital Spatial Analysis (DSP) of Tissue Microarrays (TMAs) to Study Gastric Cancer Tissue Microenvironments

Commercially available normal stomach tissue TMAs and gastric cancer biopsies were obtained from BioChain Institute Inc. (Newark, CA, USA). Tumor-derived formalin fixed paraffin embedded (FFPE) tissue sections were obtained from different subtypes of patient stomachs. The approval to collect tissue specimens was acquired from the Biochain Human Research Protections (OHRP registration number, IRB00008283) and approved by the United States Department of Health and Human Services.

The nCounter DSP barcoding technology (NanoString Technologies, Seattle, WA, USA) was applied following the manufacturer’s instructions. Briefly, FFPE TMAs were deparaffinized, subjected to antigen retrieval procedures, and incubated overnight with fluorescently-labeled PanCK, CD68 and SMA visualization antibodies and an antibody cocktail containing 40 different primary antibodies against immune, stromal and epithelial cell markers (Human Immune Cell Profiling Panel Protein Core, NanoString Technologies) [34]. Slides were scanned using the GeoMx DSP instrument (NanoString Technologies) to generate digital fluorescent images of the tissues. ROIs representing the entire TMA were selected and then segmented into epithelium/tumor, nonimmune cell stroma, as well as immune cell tissue compartments, as molecularly defined by fluorescent colocalization. Oligos from these three compartments were sequentially released upon UV exposure, collected by microcapillary aspiration, dispensed into a 96-well plate, hybridized to optical barcodes, and finally quantified by the nCounter system. Digital counts corresponding to protein markers were normalized to spike-in controls and the areas of their corresponding compartments. Compartments having an area of illumination (AOI) of less than 100 µm^2^ or 10 nuclei were automatically excluded from the analysis.

### 2.14. Statistical Analysis

Data were plotted as a mean value ± SEM. Statistical analyses using Student’s *t*-Test, One-Way ANOVA, Correlation and Regression were performed using GraphPad PRISM, to determine the differences between groups. Statistical significance was calculated based on *p* < 0.05.

## 3. Results

### 3.1. HER2 Was Significantly Expressed in the Intestinal Subtype of Gastric Cancer and Correlated with a Higher Expression of PD-L1 in the Commercial Gastric Cancer TMAs

**Figure 1:**
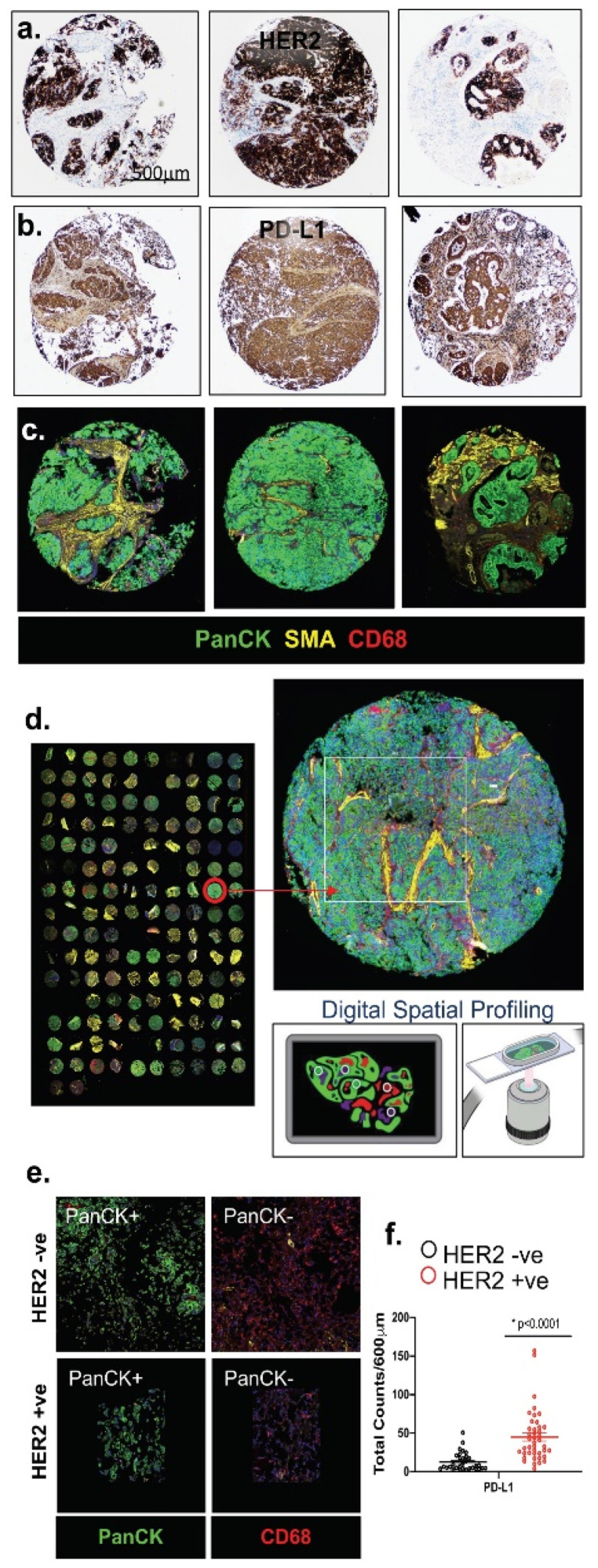
Immunohistochemistry and DSP analysis of FFPE tissue sections obtained from normal stomach and gastric tumor tissues. Immunohistochemical staining of representative ROIs for (**a**) HER2 and (**b**) PD-L1. DSP analysis of representative ROIs selected from (**c**) gastric cancer tissues showing staining for PanCK, SMA and CD68. DSP analysis of the whole tissue array (**d**) and a higher magnification of the selected tissue with ROI selection for Digital Spatial profiling and UV crosslinking. Analysis of the (**e**) tumor marker (PanCK) and immune marker (CD68) among PanCK-positive (+ve) and PanCK-negative (−ve) (CD68 + ve) areas of the FFPE tissues. (**f**) Quantitative expression of PD-L1 in HER2 −ve and HER2 +ve gastric cancer tissues. * *p* < 0.0001 compared to HER2 −ve gastric cancer tissue, *n* = 26 HER2 −ve and *n* = 46 HER2 +ve) (CD68 + ve) areas of the FFPE tissues. (**f**) Quantitative expression of PD-L1 in HER2 −ve and HER2 +ve gastric cancer tissues. * *p* < 0.0001 compared to HER2−ve gastric cancer tissue, *n* = 26 HER2 −ve and *n* = 46 HER2 +ve gastric cancer patients. Scale bar = 500 μm.

The immunohistochemistry of a gastric cancer tissue array established a direct correlation between HER2 expression and the expression of PD-L1 in the intestinal-type gastric cancer (Figure 1a,b). The NanoString DSP of tumor- and immune-cell specific protein markers using FFPE slide-mounted tissues from different subtypes of gastric cancer patients was then analyzed (Figure 1c,d). We selected 6–12 ROIs (regions of interests) within the tumor microenvironment (TME) of intestinal, diffuse and signet ring cell type gastric cancers (Figure 1c). The results showed a high level of HER2 and PD-L1 marker expression, which was significant in the intestinal-type gastric cancers within the cancer cells of the microenvironment (Figure 1e,f). These data suggested that there was a direct and significant correlation.

**Figure 2:**
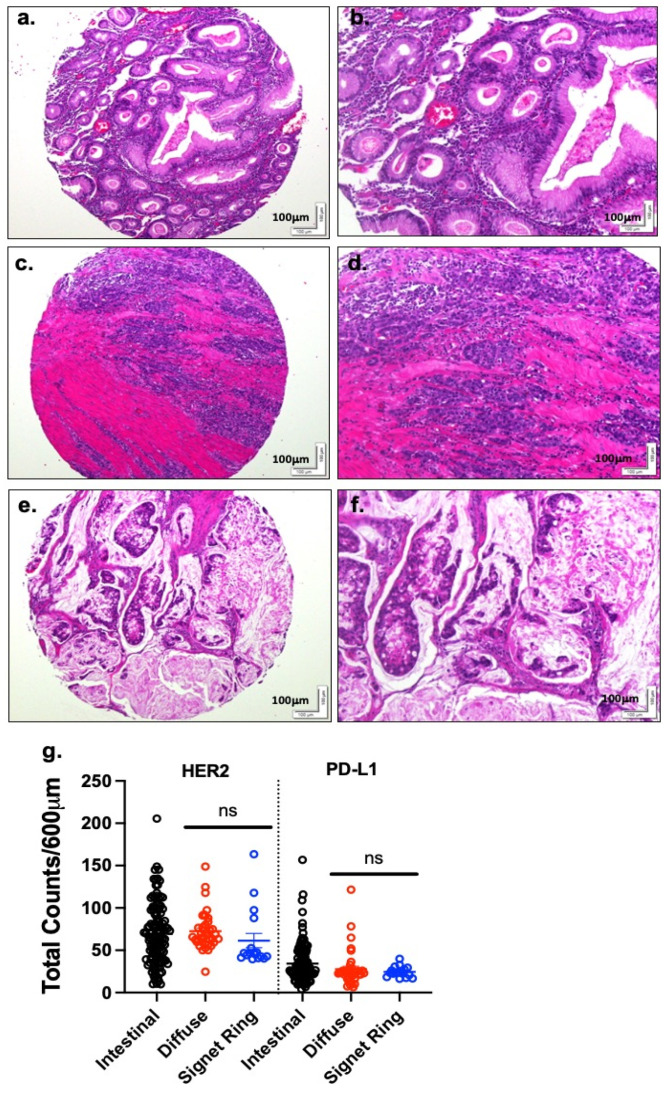
PD-L1 and HER2 expression in intestinal, diffuse and signet ring gastric cancer subtypes. H&E stain of (**a**,**b**) intestinal, (**c**,**d**) diffuse, and (**e**,**f**) signet ring gastric cancer subtypes. A higher magnification is demonstrated in panels (**b**), (**d**), and (**f**). (**g**) Quantification of HER2 and PD-L1 in the TMA using NanoString DSP. ns = not significant. Scale bar = 100 μm.

Because gastric cancers express HER2 and PD-L1 within the TME, this implies that the inhibition of HER2 may create a conducive environment for tumor immunotherapy. We quantified and compared the expression of HER2 and PD-L1 between the different subtypes of gastric cancer (Figure 2). By DSP analysis, there were no significant differences in the HER2 and PD-L1 expression between the diffuse, signet ring and intestinal subtypes. This is not surprising, as the signet ring cell subtype is classified under the Lauren classification system as a diffuse subtype of gastric cancer. In contrast, there was moderate variation in the expression of HER2 and PD-L1 between the intestinal and diffuse/signet ring cell gastric cancer subtypes, but these findings were not statistically significant. A possible reason could be the small sample size (diffuse/signet ring cell gastric cancer subtypes (*n* = 16, 10) compared to the intestinal one (*n* = 105)) in the stomach TMA (Table S1).

**Figure 3:**
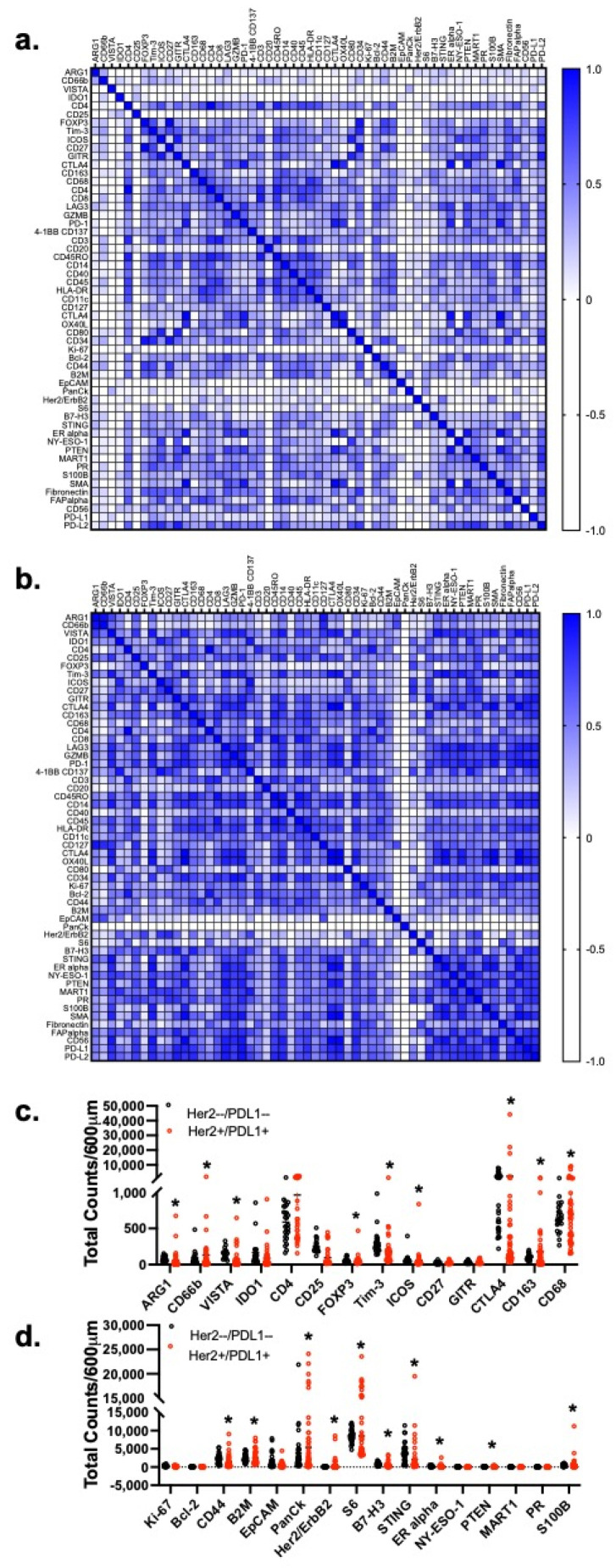
Correlation matrix of the expression of immune and tumor protein markers. Correlation matrices of the expression of immune and tumor protein markers of FFPE tissues obtained from (**a**) HER2 −ve and (**b**) HER2 +ve gastric cancer patients. Analysis of tumor cell markers (**c**) between HER2 −ve and HER2 +ve gastric cancer patients. Analysis of immunosuppressive (MDSC, Tregs and TAM) markers in (**d**) HER2 −ve and HER2 +ve gastric cancer patients. * *p* < 0.05 compared to HER2 −ve gastric cancer patients.

The correlation matrix (Figure 3a) between 55 protein markers showed a stronger association between immune-related markers and tumor markers in the HER2 and PD-L1 positive gastric cancer patient tissues (Figure 3b). The expression of certain immunosuppressive markers (FOXP3, Tim3, ICOS, CD163,) including PMN-MDSC markers (ARG1, CD66b, VISTA and IDO1) were significantly (*p* < 0.01) higher in the FFPE gastric cancer patient tissues which exhibited a higher expression of HER2 and PD-L1 (Figure 3c), when compared to HER2 and PD-L1 negative gastric cancer patient tissues. Interestingly, these immunosuppressive protein markers were also significantly (* *p* < 0.05) correlated with an increased expression of certain tumor markers, such as CD44, B2M, HER2/ErbB2, S6, B7-H3, ER alpha and S100B (Figure 3d).

### 3.2. Disruption of HER2 Signaling Drives Cancer Cell Death in Patient-Derived Gastric Cancer Organoid/Immune Cell Co-Cultures, Regardless of the Gastric Cancer Subtypes

**Figure 4:**
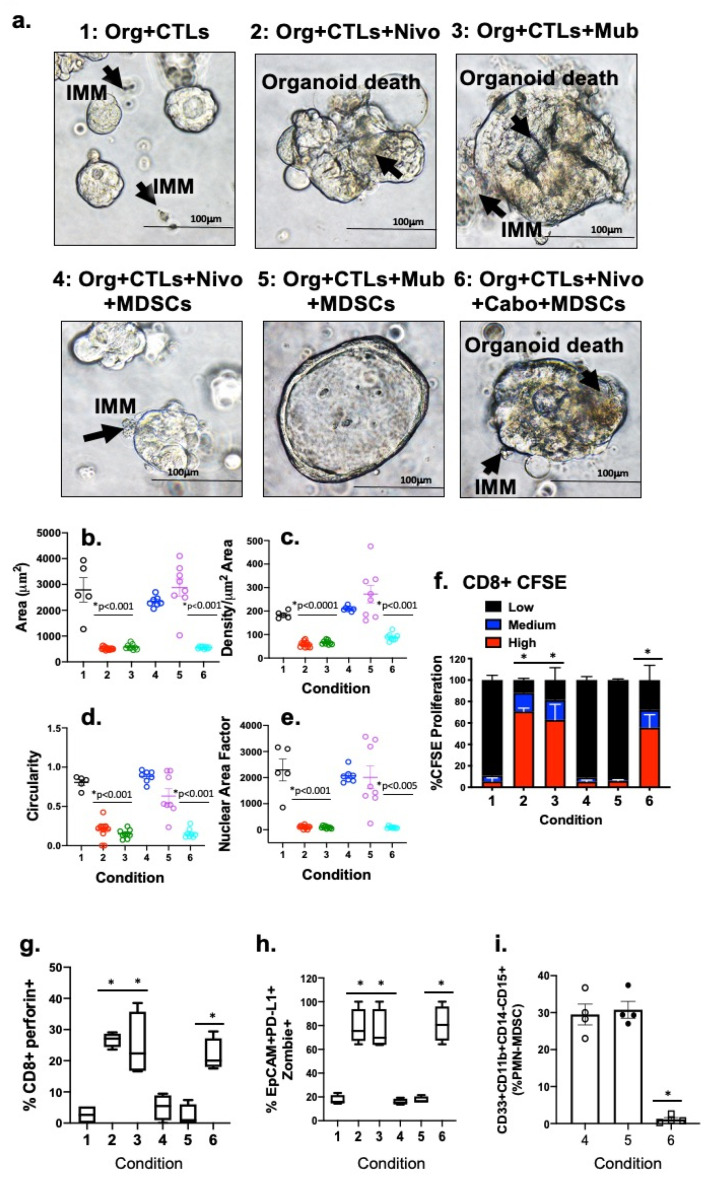
Depletion of PMN-MDSCs sensitizes PD-L1 + huTGO/immune cell (IMM) co-cultures to Nivolumab. (**a**) Morphological alterations of huTGOs 48 h after the in vitro vehicle (Control) treatment in co-culture with CTLs (Condition 1), Nivolumab (0.5 µg/mL; Condition 2) or Mubritinib (6 nM; Condition 3); in co-cultures with CTLs, Nivolumab, and MDSCs (Condition 4) or CTLs, Mubritinib, and MDSCs (Condition 5); or Nivolumab and Cabozantinib together (10 µM, Condition 6) in co-cultures with CTLs and MDSCs. Quantitative changes in (**b**) area (**c**) density (**d**) circularity and (**e**) nuclear area factor. (**f**) Quantitative changes of CTL proliferation by CFSE, shown as percent (%) determined from flow cytometry data. Changes in (**g**) expression of perforin in CTLs, (**h**) percentage of viable EpCAM+/PD-L1+ huTGOs and (**i**) PMN-MDSCs after the treatments in Conditions 4 to 6. * *p* < 0.05 compared to Condition 1, shown here are n = 4 representative examples out of a total of 11 analyzed huTGO/immune cell co-cultures. Scale bar = 100 μm.

Organoid/CTL co-cultures, established from autologous patient tissue and blood, treated with Nivolumab or Mubritinib, exhibited significant organoid death (Conditions 2 and 3 respectively, Figure 4a–e) in contrast to untreated controls (Condition 1, Figure 4a–e). When PMN-MDSCs, a commonly expressed immune suppressive cell, were added to the co-culture this response was suppressed (Conditions 4 and 5, Figure 4a–e). The addition of Cabozantinib (cabo) to the organoid/CTL/MDSC co-culture lowered the amount of MDSCs and amplified the efficacy of the checkpoint inhibition, inducing PD-L1 positive organoid death (Condition 6, Figure 4a–e). The cell death kinetics were measured by counting the organoid area (Figure 4b), density (Figure 4c) and perimeter of different regions of interest (ROIs) occupied within each co-culture. Advanced stages of apoptosis were demonstrated by calculating morphological differences, such as circularity (4 × pi × Area)/Perimeter^2^), Figure 4d) and nuclear area factor (NAF, Circularity × Area, Figure 4e). Organoid death was also enumerated by flow cytometry of Zombie (viability dye)+/EpCAM+/PD-L1+ cells (Figure 4h).

We analyzed cell CFSE uptake and CD8+ perforin expression within PMN-MDSC co-cultures to investigate the effect of PMN-MDSCs on CD8+ T cell proliferation. CD8+ T cells in gastric cancer organoid cultures without PMN-MDSCs exhibited a significant (* *p* < 0.05) rise in CTL proliferation due to Nivolumab or Mubritinib (Conditions 2 and 3 respectively, Figure 4f,g). This response was repressed with the addition of PMN-MDSCs within the co-culture (Conditions 4 and 5, Figure 4f,g). The addition of Cabozantinib with Nivolumab or Mubritinib induced CD8+ T cell proliferation (Condition 5, Figure 4f,g) which was associated with a depletion of PMN-MDSCs within Condition 6 (Figure 4i). A total of 11 gastric cancer cases of different subtypes (as listed in Table 1) were analyzed for all experimental conditions.

**Figure 5:**
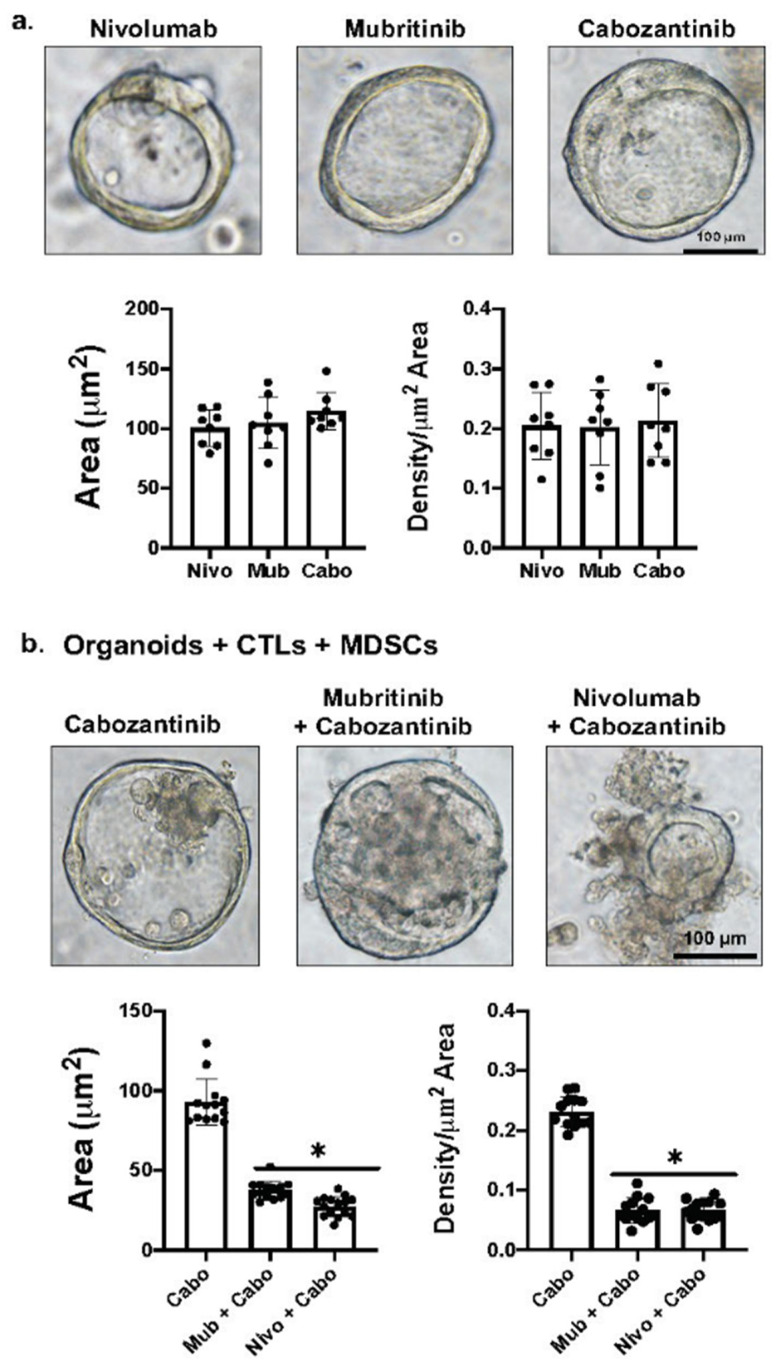
Effect of drug alone on organoid growth and viability. (**a**) Brightfield images and quantification of organoid area using cultures treated with Nivolumab, Mubritinib and Cabozantinib alone. (**b**) Brightfield images and quantification of organoid area using organoid/CTL/MDSC co-cultures treated with Cabozantinib alone, Nivolumab plus Cabozantinib, or Mubritinib plus Cabozantinib. * *p* < 0.05 compared to Cabozantinib alone. Scale bar = 100 μm.

Figure 5 demonstrates the effects of Cabozantinib alone, Nivolumab plus Cabozantinib, or Mubritinib plus Cabozantinib on the viability of organoids in the co-cultures. We observed that Cabozantinib alone has no effect on organoid viability in the presence of MDSCs, as a result of the persistent expression of PD-L1 in these cultures. It is only when Mubritinib or Nivolumab are given in combination with Cabozantinib that we observe organoid death (Figure 5).

### 3.3. Knockdown of HER2 Disrupts AKT/mTOR Signaling and Correlates with a Loss of PD-L1 Expression

**Figure 6:**
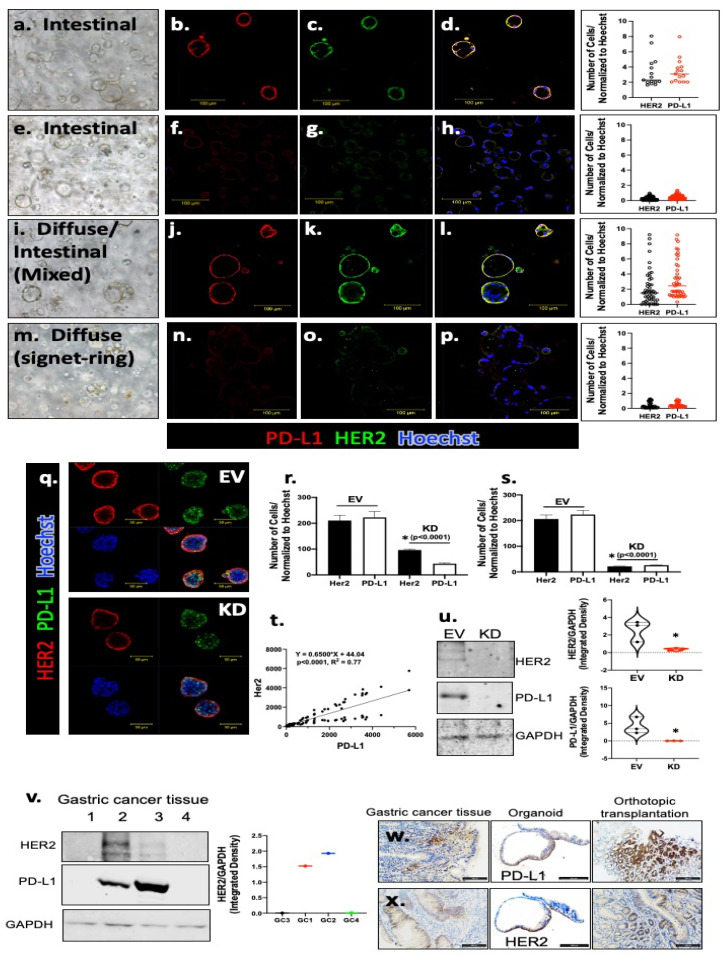
HER2 expression is significantly correlated with PD-L1 expression in gastric cancer tissues. Brightfield images showing morphological differences among huTGOs derived from intestinal (**a**,**e**) and diffuse (**i**,**m**) subtypes of gastric cancer tissues. Immunofluorescence staining of these organoids showed a high expression of (**b**–**d**,**j**–**l**) HER2 (red) and PD-L1 (green) in two of the organoid lines while others showed a negative expression (**f**–**h**,**n**–**p**) for both. Western blot (**v**) analysis of gastric cancer tissues showed a similar expression pattern. (**q**) shRNA knockdown of HER2 showed a downregulation of PD-L1 expression which was confirmed by (**u**) Western blot analysis and quantification. (**r**,**s**) Quantification of HER2 and PD-L1 positive cells showed a significant reduction in the expression of both genes after HER2 shRNA knockdown; * *p* < 0.0001. (**t**) Linear regression analysis showing a positive correlation of HER2 and PD-L1; Immunohistochemistry of PD-L1 (**w**) and HER2 (**x**) retained high expression levels among gastric cancer tissues, tissue-derived organoids and orthotopically transplanted organoids. Scale bar = 100 μm and 50 μm.

To assess the relationship between HER2 and PD-L1, lentiviral constructs containing either human shRNA targeting human HER2 (KD) or a scrambled sequence (as a negative control, EV) were used to knockdown HER2. These were applied to huTGOs that exhibited expression of HER2 and PD-L1 (Figure 6a–d,m–p). The immunofluorescence staining of four independent TGOs showed a strong association between HER2 and PD-L1 expression (Figure 6a–r). The successful knockdown of HER2 in TGO lines (Figure 6s) reduced the expression of PD-L1 in the same lines (Figure 6t,u and Figure S1).

The expression of HER2 in TGOs reflected HER2 and PD-L1 expression in the patient’s tumor tissue (Figure 6v–x). In addition, TGOs that were orthotopically transplanted into the stomachs of NSG mice, engrafted and generated human-specific HER2/PD-L1 positive tumors within the mouse gastric epithelium (Figure 6v–x).

### 3.4. Knockdown of HER2 Sensitizes Patient-Derived huTGOs to PD-1/PD-L1 Checkpoint Inhibition

**Figure 7:**
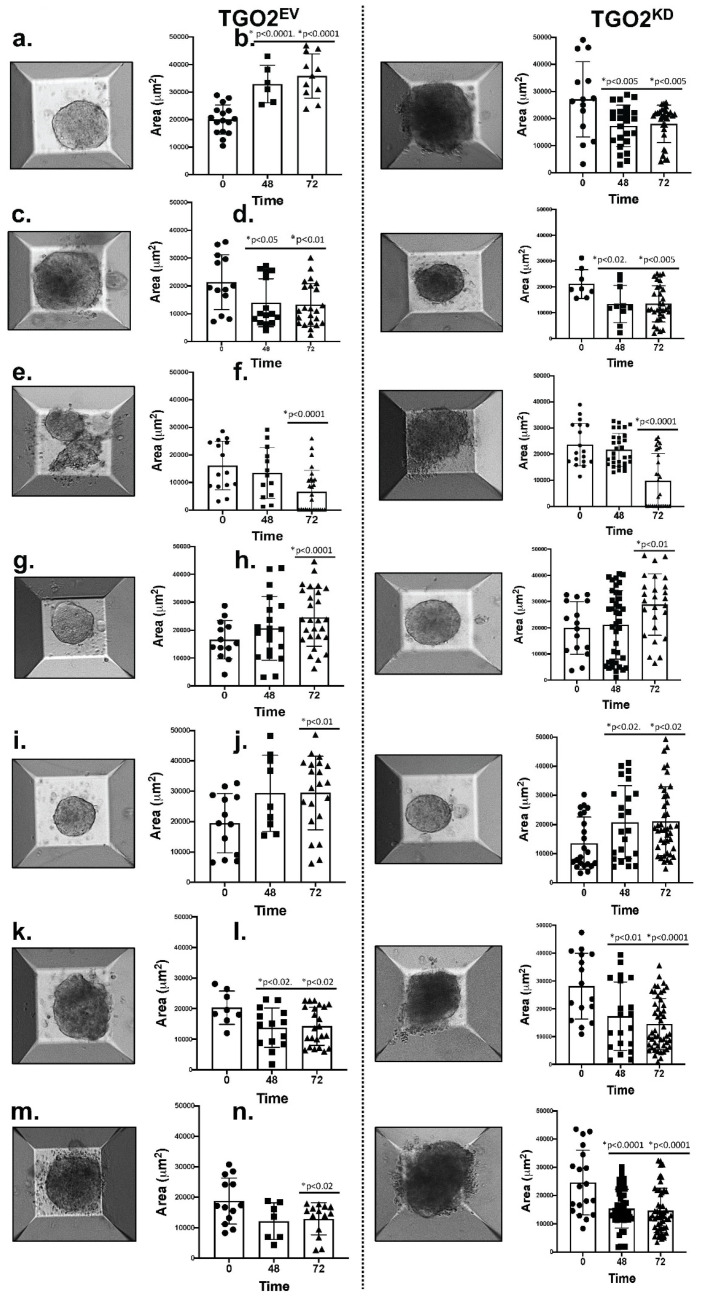
Morphological changes of EV and HER2 shRNA transduced organoids in PD-L1- huTGO/immune cell co-cultures as a result of different treatment conditions. huTGO spheroid/immune cell co-cultures were established using EV and HER2 shRNA knockdown organoids in AggreWells. Changes in the area of huTGOs were measured after 48 h of vehicle (Control) treatment in co-cultures with CTLs (**a**,**b**), Nivolumab (Condition 2, **c**,**d**) or Mubritinib (Condition 3, **e**,**f**); in co-cultures with CTLs, Nivolumab and PMN-MDSCs (Condition 4, **g**,**h**), or CTLs, Mubritinib and PMN-MDSCs (Condition 5, **i**,**j**); or in a combinatorial treatment of Nivolumab and Cabozantinib (Condition 6, **k**,**l**) or Mubritinib and Cabozantinib (Condition 7, **m**,**n**) in co-cultures with CTLs and PMN-MDSCs. * *p* < 0.05 to *p* < 0.0001 compared to 0 h time point for each condition.

The huTGO/immune cell co-cultures were evaluated using the HER2 knockdown organoid lines. AggreWell™ microwell plates comprised of an array of pyramid-shaped microwells were used to monitor morphological changes where large amounts of uniform spheroids were generated from multiple heterogeneous organoids from each individual organoid line. The area of the spheroids from different microwells was measured at 24, 48 and 72 h post treatment. A reduction of the area induced by CD8+ T cells was only observed in the HER2 shRNA knockdown co-culture (Figure 7b), and this was irrespective of the Nivolumab (Figure 7d) or Mubritinib (Figure 7f) treatment. Organoid growth was arrested in EV transduced organoids in response to Nivolumab or Mubritinib (Figure 7c,e). An increase of organoid growth was observed when PMN-MDSCs were included in the co-culture (Figure 7g–j). Combinatorial treatment of Nivolumab/Mubritinib with Cabozantinib reduced the spheroid area and depleted PMN-MDSCs within the co-culture (Figure 7k–n).

### 3.5. Knockdown of HER2 Results in the Loss of AKT-mTOR Signaling as Well as a Decrease in PD-L1 Expression in Patient-Derived Gastric Cancer Organoids

**Figure 8:**
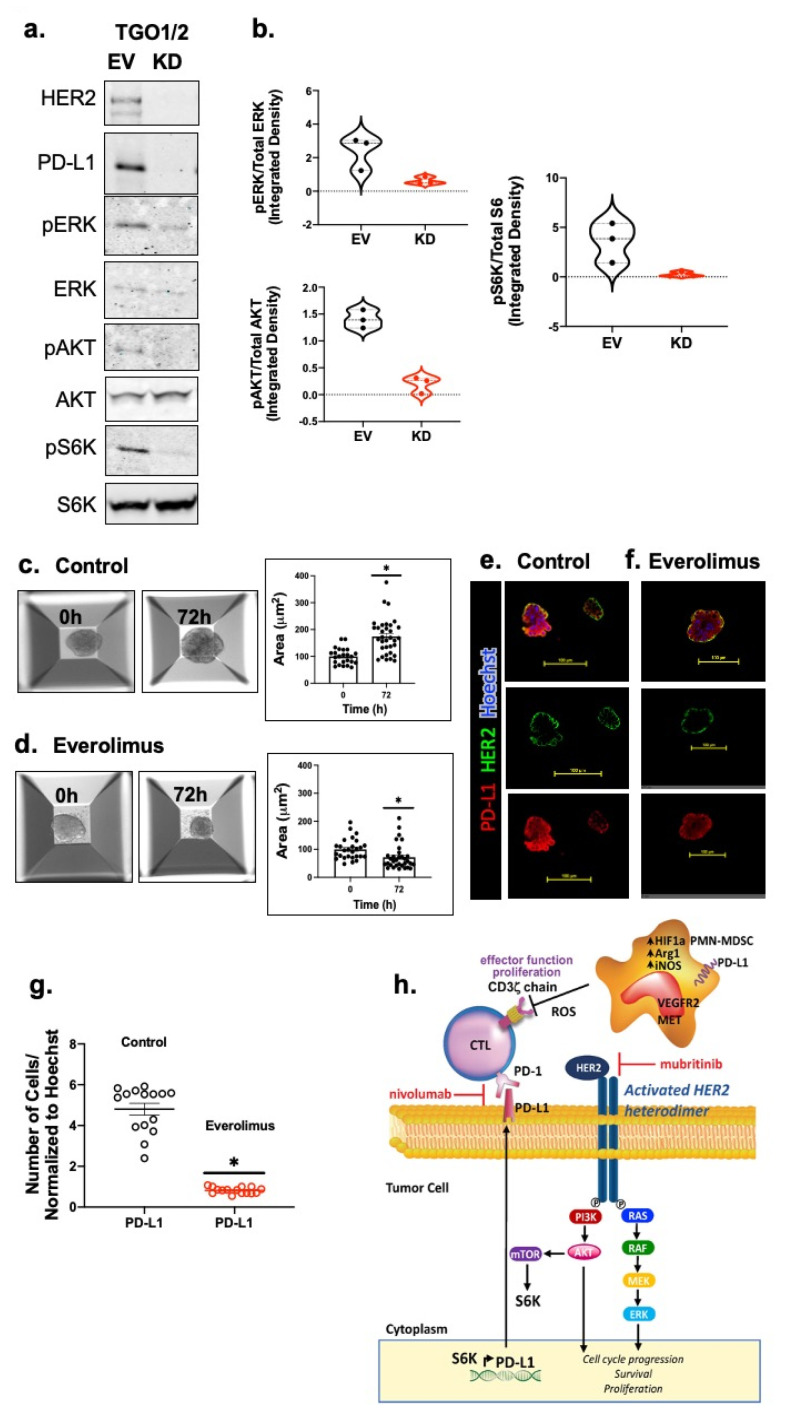
Inhibition of HER2 regulates PD-L1 expression by suppressing HER2/AKT/MTOR signaling in gastric cancer-derived organoids. The expression of the HER2/AKT/MTOR signaling pathway components were determined by (**a**) Western blot analysis. (**b**) Violin plots representing densitometric values of protein bands measured from 8a. Brightfield images and the calculated organoid area in cultures treated with (**c**) vehicle (control), or (**d**) Everolimus. n = 15 ROI from 3 different experiments, * *p* < 0.05 in comparison to vehicle. Immunofluorescences staining using antibodies specific for HER2 (green) and PD-L1 (red) using (**e**) vehicle (control), or (**f**) Everolimus treated organoid cultures. (**g**) Quantification of fluorescence intensity for PD-L1 expression in the control or Everolimus treated cultures. (**h**) Proposed mechanisms of anti-PD1 inhibition and suppression of MDSCs in a combination immunotherapy strategy for gastric cancer patients. Within the gastric TME, PMN-MDSCs override the checkpoint inhibition by releasing Arg1, iNOS and ROS. Pharmacological inhibition of ERBB2/HER2 could diminish PD-L1 +ve cells from the TME, improving patient responses to immunotherapy. Scale bar = 100 μm.

In order to test whether PD-L1 expression was dependent on active PI3K–AKT–mTOR signaling, human TGO^EV^ and TGO^KD^ organoid lines were used. Western blot analysis revealed that knockdown of HER2 resulted in a disruption of ERK, AKT and S6K protein phosphorylation (Figure 8a,b and Figure S2) and this correlated with a decrease in PD-L1 expression. We also tested the effect of the mTOR inhibitor Everolimus on PD-L1 expression. Everolimus significantly lowered the expression of PD-L1 in HER2 + PD-L1 + patient-derived organoids (Figure 8e–g) and this correlated with a significant inhibition of organoid growth and viability as measured by the area (Figure 8c,d). Our data confirmed that the inhibition of mTOR significantly decreased the expression of PD-L1 in patient-derived organoids (*n* = 9 out of 11 total gastric cancer cases which were positive in both HER2 and PD-L1 as listed in Table 1). Collectively, these data demonstrate that knockdown of HER2 results in the inhibition of AKT-mTOR signaling, resulting in a reduced PD-L1 expression.

## 4. Discussion

We have previously reported the effects of anti-tumor agents on gastric cancer patient-derived organoids generated from resected specimens [29]. In this study, we described the successful establishment of patient-derived organoids from biopsied tissues, which are on average only 3–5 mm in diameter. This opens up multiple opportunities for testing precision medicine in vitro, where sampling of tissue specimens can be conducted simply via endoscopy and may be beneficial for patients who are not suitable to undergo surgical procedures.

Here, we illustrated a higher PD-L1 expression in gastric cancer organoids derived from patients co-expressing HER2, as compared to those that are HER2-negative. Attenuation of the HER2 pathway decreased PD-L1 expression and this correlated with a loss of inhibition of AKT-mTOR signaling. Using Digital Spatial Profiling (DSP) to evaluate the protein expression in tumor tissue and the surrounding stroma and immune cells, we detected a direct association between increased HER2 and PD-L1 expression. The status of the HER2 gene often determines the selection and responsiveness of a targeted treatment for patients with advanced disease [35]. Currently, the detection of the HER2 gene in advanced gastric cancer is routine in clinical pathology, and many countries have generated HER2 detection guidelines [35]. Importantly, the PD-1/PD-L pathway is critical for immunosuppression. Using patient gastric cancer tissue for DSP analysis and organoid cultures derived from HER2-positive and -negative gastric cancer patients, our present study documents that HER2 and PD-L1 were jointly detected in gastric cancer. Our data is strongly supported by published studies that have performed extensive immunohistochemical and fluorescence in situ hybridization to show that HER2 and PD-L1 are not only co-expressed but related to the gastric cancer stage and lymph node metastasis [36,37,38,39,40,41]. We used autologous gastric cancer patient-derived organoid/immune cell co-culture models [30] to identify the mechanisms by which HER2 regulates the expression of PD-L1 in gastric cancer. Thus, our studies may provide an important reference for the benefit of targeted combinatorial therapy for gastric cancer treatment.

The immune cell compartment of HER2/PD-L1 positive gastric cancer shows the highest infiltration of CD68-positive immune markers versus negative compartments [37]. CD68-positive monocyte cells highly express immune suppressive proteins, including the most common polymorphonuclear myeloid-derived suppressor cells (PMN-MDSCs) which express Arg1, CD66b, VISTA and IDO1. Concomitant with our findings, a poor prognosis and reduced survival of advanced gastric cancer are correlated with an increased CD33+CD11b+CD15+CD14-expressing PMN-MDSC infiltration [42]. PMN-MDSCs contribute to immune suppressive mechanisms by blocking the CD8+ T cell effector function via either the sequestration of L-arginine and L-cysteine or the production of ROS [42,43]. Importantly, recent studies have documented, while responding to an *H. pylori* infection, the infiltration of a unique subset of PMN-MDSCs which express MIR130B, an endogenous short noncoding RNA, as well as TNF-α, known to contribute to immunotherapy-resistant gastric cancer [44,45]. Consistent with the importance of PMN-MDSCs in the progression of gastric cancer, we observed these immune cells significantly inhibited the efficacy of Nivolumab- or Mubritinib-induced tumor cell death by inhibiting CTL proliferation and effector function in the organoid/immune cell co-cultures. Clinical trials researching breast, prostate, and renal cancers demonstrated that the tyrosine kinase inhibitor Cabozantinib inhibits the immunosuppressive function of MDSCs [46,47]. Our findings revealed that the depletion of PMN-MDSCs through Cabozantinib in co-cultures sensitized gastric cancer organoids to both Nivolumab and Mubritinib. Studies using gastric cancer cell lines have clearly demonstrated that the inhibition of HER2 overexpression leads to a decrease in PD-L1 expression and may thereby create an environment conducive for tumor immunotherapy [36]. However, these studies did not include combinatorial therapy in the context of immune suppressive cells. The effectiveness of targeting MDSCs together with immunotherapy has yet to be determined for the treatment of gastric cancer patients.

Knockdown of HER2 significantly reduced the phosphorylation of AKT, ERK and S6K proteins and this correlated with tumor cell death and an increased CTL effector function. Both PI3K-AKT-mTOR and RAS-RAF-MEK pathways are downstream of EGFR/HER2 signaling [36,48,49]. In fact, findings using gastric cancer cell lines showed that HER2-mediated PD-L1 expression is via the PI3K-AKT-mTOR pathway (Figure 8h), and inhibition of the PI3K–AKT–mTOR pathway decreases PD-L1 expression [36]. Hedgehog (Hh) signaling is another pathway shown to regulate the expression of PD-L1 in gastric cancer [50,51]. Research has demonstrated that inhibiting Hh signaling using GANT61 (Gli-inhibitor) decreases PD-L1 expression and tumor proliferation, but increases the infiltration of CD8+ CTLs both in vivo in a gastric cancer mouse model, and in vitro in gastric cancer patient-derived organoid cultures [52,53]. These findings are of significance given that SLFN + PMN-MDSCs are a transcriptional target of GLI1, the downstream signaling molecule of the canonical Hh pathway regulating PD-L1 expression [44,45]. Atypical activation of GLI1 has been observed in esophageal adenocarcinoma (EAC) via the mTOR/S6K1 pathway which activates the transcription and oncogenic function of GLI1 phosphorylation by S6K1 [54]. Taken together, these studies suggest a plausible connection between mTOR/S6K1 and Hh signaling in regulating PD-L1 expression.

To further ascertain that disruption of HER2-induced PD-L1 expression abolishes tumor cell immune evasion, organoid models of different cancer types can be similarly analyzed. For instance, overexpression of HER2 is common in breast cancer cases. Breast cancer patient-derived organoids with HER2 amplification may act as a separate ex vivo model system to substantiate our findings from the gastric cancer organoid model reported in this study.

HER2-positive gastric cancer exhibits a poor prognosis. The median survival is 21 months (range 10–57 months) and the 5-year survival rate is 42% [55,56]. Trastuzumab has been used in clinics along with the standard of care chemotherapeutic drugs like 5FU, Capecitabine or Cisplatin for advanced stage gastric cancers [20]. Although approximately 40% of gastric tumors express PD-L1, only 30% of these patients are responders [57]. Clinical studies have shown a more effective combinatorial antitumor treatment modality with Margetuximab, which targets HER2, and Pembrolizumab, which targets PD-1, in patients with HER2-positive gastro-esophageal adenocarcinoma [58].

## 5. Conclusions

In the present research, we demonstrate that HER2-induced PD-L1 may drive tumor-immune cell evasion. As we previously reported [30], organoid/immune cell co-cultures may be used to effectively screen for targeted therapeutic approaches. Here, our findings suggest that drugs targeting HER2 could inhibit CTL effector functions and PD-L1 expression through the PI3K-AKT-mTOR pathway. HER2 inhibition may therefore create a milieu conducive to effective immunotherapy for the treatment of gastric cancer.

## Figures and Tables

**Table 1:** Demographics and Pathological Profiles of Gastric Cancer Patients.

Patient ID	Sex	Age	Subtype	Histological Grade	Stage (TNM)	HER2 Expression	PD-L1 Expression
SC046	F	67	Intestinal	Moderately to poorly differentiated	T3N0M0	Positive	Positive
SC047	M	59	Intestinal	Moderately differentiated	T3N3bMx	Positive	Positive
SC049	M	36	Intestinal	Moderately differentiated	T4bN3b	Positive	Positive
SC052	F	66	Diffuse	Poorly differentiated	T1bN0	Positive	Positive
SC054	F	85	Intestinal	Well differentiated	T3N2	Positive	Positive
SC055	M	74	Mixed	Moderately differentiated	T4aN3a	Positive	Positive
SC061	M	84	Intestinal	Well differentiated	T1bN0	Negative	Positive
huTGO1	F	65	Mixed	Poorly differentiated	T3N1M1	Positive	Positive
huTGO2	F	82	Intestinal	Well differentiated	T1bN0M0	Positive	Positive
huTGO4	M	56	Diffuse	Poorly differentiated	T4N3M0	Positive	Positive
huTGO5	M	75	Intestinal	Moderately differentiated	T3N0M0	Negative	Negative

**Table 2:** Composition of gastric organoid growth medium.

Reagents	Final Concentration	Manufacturer
Advanced DMEM/F12		Thermo Fisher Scientific Waltham, MA, USA
Penicillin/Streptomycin	1%	Thermo Fisher Scientific Waltham, MA, USA
Hepes	10 mM	Thermo Fisher Scientific Waltham, MA, USA
Glutamax	1×	Thermo Fisher Scientific Waltham, MA, USA
B27	1×	Thermo Fisher Scientific Waltham, MA, USA
N2	1×	Thermo Fisher Scientific Waltham, MA, USA
Nicotinamide	10 mM	Sigma-Aldrich St. Louis, MI, USA
N-Acetyl cysteine	1 mM	Sigma-Aldrich St. Louis, MI, USA
Y-27632	10 µM	Sigma-Aldrich St. Louis, MI, USA
Noggin	100 ng/mL	Peprotech Cranbury, NJ, USA
FGF-10	200 ng/mL	Peprotech Cranbury, NJ, USA
EGF	50 ng/mL	Peprotech Cranbury, NJ, USA
Gastrin 1	1 nM	TOCRIS Bioscience
Wnt Conditioned Medium	50%	
R-Spondin Conditioned Medium	10%	

## Data Availability

The datasets generated during and/or analyzed during the current study are available in the ReDATA repository, https://data.library.arizona.edu/services/research-data-repository-redata#exceptions (accessed on 10 January 2021). The datasets generated during and/or analyzed during the current study are also available from the corresponding author on reasonable request. All data generated or analyzed during this study are included in this published article (and its Supplementary Information Files).

## Supplementary Materials

The following are available online at https://www.mdpi.com/article/10.3390/cancers13246158/s1, Table S1: Stomach Tumor Tissue MicroArray (TMA) used for DSP Analysis. Figures S1 and S2.

## Abbreviations

CFSE	Carboxyfluorescein succinimidyl ester
CTL	Cytotoxic T- Lymphocyte
DC	Dendritic Cell
DMEM/F12	Dulbecco’s Modified Eagle Medium/Nutrient Mixture F-12
DPBS	Dulbecco’s Phosphate-Buffered Saline
DSP	Digital Spatial Profiling
EV	Empty Vector
FCS	Fetal Bovine Serum
FGF-10	Fibroblast Growth Factor 10
GMCSF	Granulocyte-Macrophage Colony Stimulating Factor
HBSS	Hank’s Balanced Salt Solution
HER2	Human epidermal growth factor receptor 2
huTGO	Human gastric tumor-derived organoids
IDO-1	Indoleamine 2,3-Dioxygenase 1
KD	Knock Down
MDSC	Myeloid-Derived Suppressor Cell
NAF	Nuclear area factor
NSG	NOD Scid Gamma
PD-1	Program Death 1
PD-l1	Program Death Ligand 1
PMN-MDSC	Polymorphonuclear MDSC
ROI	Region of Interest
ROS	Reactive Oxygen Species
SMA	Smooth Muscle Actin
TAM	Tumor-associated macrophages
TGFβ	Transforming Growth Factor beta
TMA	Tissue microarrays
TME	Tumor Microenvironment
Treg	Regulatory T cell
VEGF	Vascular Endothelial Growth Factor
VISTA	V-domain Ig Suppressor of T Cell Activation
