# Supplementary material for: Disruption of Her2-Induced PD-L1 Inhibits Tumor Cell Immune Evasion in Patient-Derived Gastric Cancer Organoids
Source: Cancers (Basel). 2021 Dec 7;13(24):6158. doi: 10.3390/cancers13246158 (PMC8699100; doi:10.3390/cancers13246158)
Supplement: Supplementary file 1 [file cancers-13-06158-s001.zip › cancers-1362024-supplementary.pdf]

cancers

MDPI

Supplementary Materials

Disruption of Her2-Induced PD-L1 Inhibits Tumor Cell Immune Evasion in Patient-Derived Gastric Cancer Organoids

Jayati Chakrabarti [1,†], Vivien Koh [2,3,†], Nina Steele [4,5], Jennifer Hawkins [6], Yoshiaki Ito [3], Juanita L. Merchant [7], Jiang Wang [8], Michael A. Helmrath [6], Syed A Ahmad [9], Jimmy Bok Yan So [2,10], Wei Peng Yong [2,3,*] and Yana Zavros [1,*]

Supplemental Figure S1

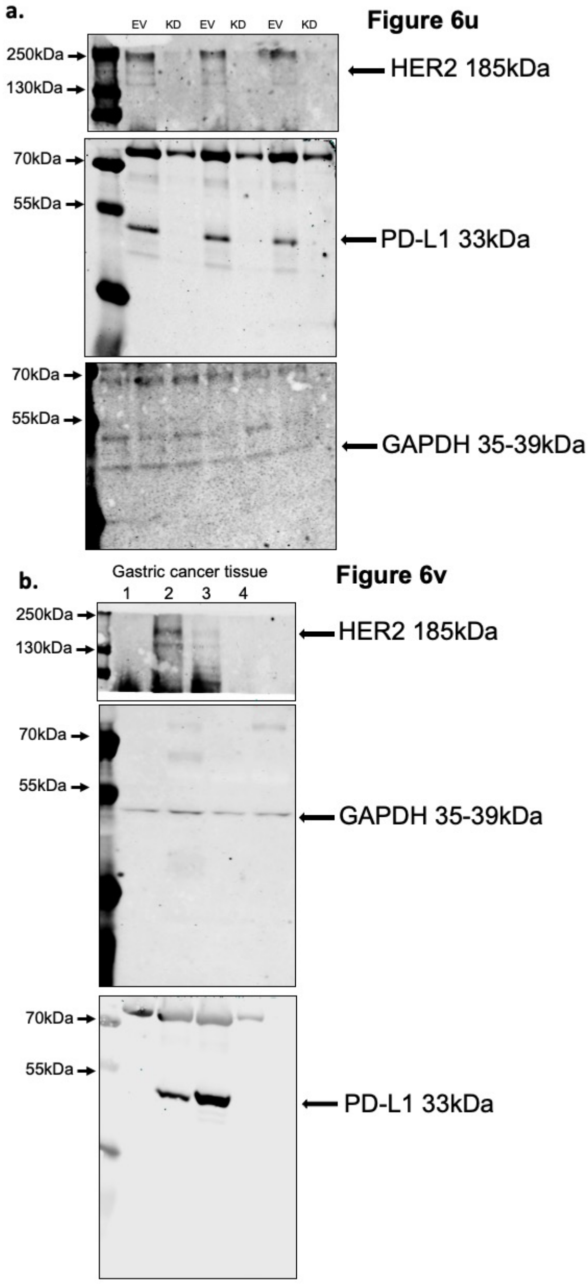

Supplemental Figure S2

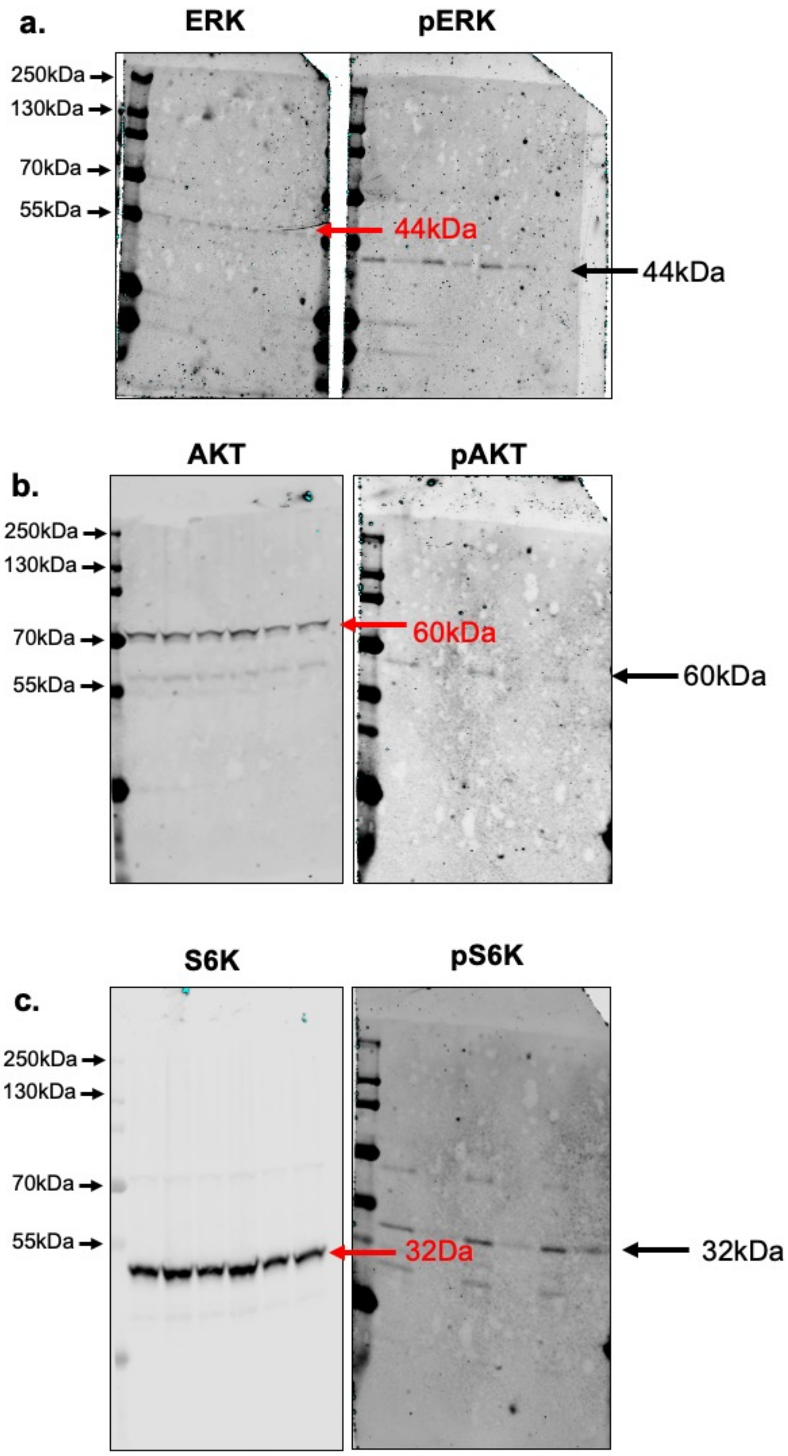

Table S1. Stomach Tumor Tissue MicroArray (TMA) used for DSP Analysis.

Position	Sex	Age	Subtype	Pathology	Nature	Grade	Stage (TNM)
**A06**	M	47	Intestinal	Adenocarcinoma	Malignant	III	T3N0M0
**A07**	M	65	Intestinal	Adenocarcinoma	Malignant	II	T3N1M0
**A08**	M	70	Intestinal	Adenocarcinoma	Malignant	II~III	T2N1M0
**A09**	M	56	Intestinal	Adenocarcinoma	Malignant	II~III	T2N1M0
**A10**	M	66	Intestinal	Adenocarcinoma	Malignant	III	T2N0M0
**A11**	F	49	Intestinal	Adenocarcinoma	Malignant	I~II	T2N0M0
**A12**	M	57	Intestinal	Adenocarcinoma	Malignant	III	T4N1M0
**A13**	F	40	Intestinal	Adenocarcinoma	Malignant	I	T3N1M0
**A14**	M	28	Intestinal	Adenocarcinoma	Malignant	III	T2N2M0
**A15**	M	70	Intestinal	Adenocarcinoma	Malignant	III	T3N1M0
**B06**	M	47	Intestinal	Adenocarcinoma	Malignant	III	T3N0M0
**B07**	M	65	Intestinal	Adenocarcinoma	Malignant	II	T3N1M0
**B08**	M	70	Intestinal	Adenocarcinoma	Malignant	II~III	T2N1M0
**B09**	M	56	Intestinal	Adenocarcinoma	Malignant	II~III	T2N1M0
**B10**	M	66	Intestinal	Adenocarcinoma	Malignant	III	T2N0M0
**B11**	F	49	Intestinal	Adenocarcinoma	Malignant	I~II	T2N0M0
**B12**	M	57	Intestinal	Adenocarcinoma	Malignant	III	T4N1M0
**B13**	F	40	Intestinal	Adenocarcinoma	Malignant	I	T3N1M0
**B14**	M	28	Intestinal	Adenocarcinoma	Malignant	III	T2N2M0
**B15**	M	70	Intestinal	Adenocarcinoma	Malignant	III	T3N1M0
**C01**	M	35	Signet Ring/Diffuse	Signet ring cell carcinoma	Malignant		T3N0M0
**C02**	F	44	Intestinal	Adenocarcinoma	Malignant	III	T3N0M0
**C03**	F	50	Intestinal	Adenocarcinoma	Malignant	III	T3N1M0
**C04**	M	57	Intestinal	Adenocarcinoma	Malignant	III	T3N1M0
**C05**	M	48	Intestinal	Adenocarcinoma	Malignant	I~II	T3N1M0
**C06**	M	58	Intestinal	Adenocarcinoma	Malignant	I~II	T2N1M0
**C07**	M	72	Intestinal	Adenocarcinoma	Malignant	III	T3N2M0
**C08**	M	53	Intestinal	Adenocarcinoma	Malignant	III	T3N1M0
**C09**	M	60	Intestinal	Adenocarcinoma	Malignant	III	T3N1M0
**C10**	M	62	Intestinal	Adenocarcinoma	Malignant	II~III	T3N1M0
**C11**	M	61	Intestinal	Adenocarcinoma	Malignant	III	T3N0M0
**C12**	F	70	Diffuse	Undifferentiated carcinoma	Malignant		T2N1M0
**C14**	F	48	Intestinal	Adenocarcinoma	Malignant	II~III	T2N0M0
**C15**	M	47	Signet Ring/Diffuse	Signet ring cell carcinoma	Malignant		T3N2M0
**D01**	M	35	Signet Ring/Diffuse	Signet ring cell carcinoma	Malignant		T3N0M0

**D02**	F	44	Intestinal	Adenocarcinoma	Malignant	III	T3N0M0
**D03**	F	50	Intestinal	Adenocarcinoma	Malignant	III	T3N1M0
**D04**	M	57	Intestinal	Adenocarcinoma	Malignant	III	T3N1M0
**D05**	M	48	Intestinal	Adenocarcinoma	Malignant	I~II	T3N1M0
**D06**	M	58	Intestinal	Adenocarcinoma	Malignant	I~II	T2N1M0
**D07**	M	72	Intestinal	Adenocarcinoma	Malignant	III	T3N2M0
**D08**	M	53	Intestinal	Adenocarcinoma	Malignant	III	T3N1M0
**D09**	M	60	Intestinal	Adenocarcinoma	Malignant	III	T3N1M0
**D10**	M	62	Intestinal	Adenocarcinoma	Malignant	II~III	T3N1M0
**D11**	M	61	Intestinal	Adenocarcinoma	Malignant	III	T3N0M0
**D12**	F	70	Diffuse	Undifferentiated carcinoma	Malignant		T2N1M0
**D14**	F	48	Intestinal	Adenocarcinoma	Malignant	II~III	T2N0M0
**D15**	M	47	Signet Ring/Diffuse	Signet ring cell carcinoma	Malignant		T3N2M0
**E01**	M	60	Intestinal	Adenocarcinoma	Malignant	II~III	T2N0M0
**E02**	M	57	Intestinal	Adenocarcinoma	Malignant	II	T3N1M0
**E03**	F	62	Intestinal	Adenocarcinoma	Malignant	II~III	T2N1M0
**E04**	M	65	Intestinal	Adenocarcinoma	Malignant	I~II	T3N2M0
**E05**	M	54	Intestinal	Adenocarcinoma	Malignant	I~II	T3N0M0
**E06**	F	70	Intestinal	Adenocarcinoma	Malignant	III	T3N2M0
**E07**	M	65	Diffuse	Undifferentiated carcinoma	Malignant		T3N2M0
**E09**	F	50	Diffuse	Undifferentiated carcinoma	Malignant		T3N1M0
**E10**	M	60	Intestinal	Adenocarcinoma	Malignant	III	T2N0M0
**E11**	F	39	Intestinal	Adenocarcinoma	Malignant	II~III	T3N2M0
**E12**	M	92	Signet Ring/Diffuse	Signet ring cell carcinoma	Malignant		T3N1M0
**E13**	M	54	Intestinal	Adenocarcinoma	Malignant	III	T3N1M0
**E14**	M	60	Intestinal	Adenocarcinoma	Malignant	III	T2N1M0
**E15**	M	63	Intestinal	Adenocarcinoma	Malignant	III	T2N0M0
**F01**	M	60	Intestinal	Adenocarcinoma	Malignant	II~III	T2N0M0
**F02**	M	57	Intestinal	Adenocarcinoma	Malignant	II	T3N1M0
**F03**	F	62	Intestinal	Adenocarcinoma	Malignant	II~III	T2N1M0
**F04**	M	65	Intestinal	Adenocarcinoma	Malignant	I~II	T3N2M0
**F05**	M	54	Intestinal	Adenocarcinoma	Malignant	I~II	T3N0M0
**F06**	F	70	Intestinal	Adenocarcinoma	Malignant	III	T3N2M0
**F07**	M	65	Diffuse	Undifferentiated carcinoma	Malignant		T3N2M0

**F09**	F	50	Diffuse	Undifferentiated carcinoma	Malignant		T3N1M0
**F10**	M	60	Intestinal	Adenocarcinoma	Malignant	III	T2N0M0
**F11**	F	39	Intestinal	Adenocarcinoma	Malignant	II~III	T3N2M0
**F12**	M	92	Signet Ring/Diffuse	Signet ring cell carcinoma	Malignant		T3N1M0
**F13**	M	54	Intestinal	Adenocarcinoma	Malignant	III	T3N1M0
**F14**	M	60	Intestinal	Adenocarcinoma	Malignant	III	T2N1M0
**F15**	M	63	Intestinal	Adenocarcinoma	Malignant	III	T2N0M0
**G01**	M	52	Intestinal	Adenocarcinoma	Malignant	III	T3N1M0
**G02**	M	61	Intestinal	Adenocarcinoma	Malignant	III	T3N2M0
**G03**	M	73	Intestinal	Adenocarcinoma	Malignant	III	T2N1M0
**G04**	M	76	Intestinal	Adenocarcinoma	Malignant	II~III	T3N0M0
**G05**	M	70	Intestinal	Adenocarcinoma	Malignant	II	T2N1M0
**G06**	M	46	Diffuse	Undifferentiated carcinoma	Malignant		T3N1M0
**G07**	F	71	Intestinal	Adenocarcinoma	Malignant	III	T3N2M0
**G08**	M	66	Intestinal	Adenocarcinoma	Malignant	I	T3N1M0
**G10**	M	43	Intestinal	Adenocarcinoma	Malignant	II~III	T3N1M0
**G11**	M	44	Diffuse	Undifferentiated carcinoma	Malignant		T3N1M0
**G12**	M	58	Diffuse	Undifferentiated carcinoma	Malignant		T3N2M0
**G13**	M	44	Intestinal	Adenocarcinoma	Malignant	II	T3N0M0
**G14**	M	52	Diffuse	Undifferentiated carcinoma	Malignant		T3N0M0
**G15**	M	62	Diffuse	Undifferentiated carcinoma	Malignant		T2N0M0
**H01**	M	52	Intestinal	Adenocarcinoma	Malignant	III	T3N1M0
**H02**	M	61	Intestinal	Adenocarcinoma	Malignant	III	T3N2M0
**H03**	M	73	Intestinal	Adenocarcinoma	Malignant	III	T2N1M0
**H04**	M	76	Intestinal	Adenocarcinoma	Malignant	II~III	T3N0M0
**H05**	M	70	Intestinal	Adenocarcinoma	Malignant	II	T2N1M0
**H06**	M	46	Diffuse	Undifferentiated carcinoma	Malignant		T3N1M0
**H07**	F	71	Intestinal	Adenocarcinoma	Malignant	III	T3N2M0
**H08**	M	66	Intestinal	Adenocarcinoma	Malignant	I	T3N1M0
**H10**	M	43	Intestinal	Adenocarcinoma	Malignant	II~III	T3N1M0
**H11**	M	44	Diffuse	Undifferentiated carcinoma	Malignant		T3N1M0

**H12**	M	58	Diffuse	Undifferentiated carcinoma	Malignant		T3N2M0
**H13**	M	44	Intestinal	Adenocarcinoma	Malignant	II	T3N0M0
**H14**	M	52	Diffuse	Undifferentiated carcinoma	Malignant		T3N0M0
**H15**	M	62	Diffuse	Undifferentiated carcinoma	Malignant		T2N0M0
**I01**	F	55	Intestinal	Adenocarcinoma	Malignant	III	T3N1M0
**I02**	M	49	Intestinal	Adenocarcinoma	Malignant	III	T3N1M0
**I03**	M	61	Intestinal	Adenocarcinoma	Malignant	II~III	T3N1M0
**I04**	M	45	Intestinal	Adenocarcinoma	Malignant	III	T3N2M0
**I05**	M	53	Intestinal	Adenocarcinoma	Malignant	II	T3N2M0
**I06**	M	50	Signet Ring/Diffuse	Signet ring cell carcinoma	Malignant		T2N1M0
**I07**	F	45	Intestinal	Adenocarcinoma	Malignant	II	T3N1M0
**I08**	F	45	Intestinal	Adenocarcinoma	Malignant	III	T3N0M0
**I09**	M	65	Intestinal	Adenocarcinoma	Malignant	III	T3N1M0
**I10**	F	37	Intestinal	Adenocarcinoma	Malignant	III	T3N0M0
**I12**	M	59	Signet Ring/Diffuse	Signet ring cell carcinoma	Malignant		T3N0M0
**I13**	F	59	Intestinal	Adenocarcinoma	Malignant	III	T3N2M0
**I14**	M	62	Intestinal	Adenocarcinoma	Malignant	III	T3N0M0
**I15**	M	68	Intestinal	Adenocarcinoma	Malignant	II	T3N0M0
**J01**	F	55	Intestinal	Adenocarcinoma	Malignant	III	T3N1M0
**J02**	M	49	Intestinal	Adenocarcinoma	Malignant	III	T3N1M0
**J03**	M	61	Intestinal	Adenocarcinoma	Malignant	II~III	T3N1M0
**J04**	M	45	Intestinal	Adenocarcinoma	Malignant	III	T3N2M0
**J05**	M	53	Intestinal	Adenocarcinoma	Malignant	II	T3N2M0
**J06**	M	50	Signet Ring/Diffuse	Signet ring cell carcinoma	Malignant		T2N1M0
**J07**	F	45	Intestinal	Adenocarcinoma	Malignant	II	T3N1M0
**J08**	F	45	Intestinal	Adenocarcinoma	Malignant	III	T3N0M0
**J09**	M	65	Intestinal	Adenocarcinoma	Malignant	III	T3N1M0
**J10**	F	37	Intestinal	Adenocarcinoma	Malignant	III	T3N0M0
**J12**	M	59	Signet Ring/Diffuse	Signet ring cell carcinoma	Malignant		T3N0M0
**J13**	F	59	Intestinal	Adenocarcinoma	Malignant	III	T3N2M0
**J14**	M	62	Intestinal	Adenocarcinoma	Malignant	III	T3N0M0
**J15**	M	68	Intestinal	Adenocarcinoma	Malignant	II	T3N0M0

Information in this table was retrieved from the manufacturer's website (www.biochain.com) for the Stomach Tumor TMA catalog #Z7020046.
